# PEG-Plasma Hydrogels Increase Epithelialization Using a Human Ex Vivo Skin Model

**DOI:** 10.3390/ijms19103156

**Published:** 2018-10-13

**Authors:** Randolph Stone, John T. Wall, Shanmugasundaram Natesan, Robert J. Christy

**Affiliations:** Combat Trauma and Burn Injury Research, US Army Institute of Surgical Research, JBSA Fort Sam Houston, San Antonio, TX 78234-6315, USA; randolph.stone4.ctr@mail.mil (R.S.II); john.t.wall40.ctr@mail.mil (J.T.W.); shanmugasundaram.natesan.ctr@mail.mil (S.N.)

**Keywords:** ex vivo, epithelialization, keratinocyte, discarded skin, wound closure, biomaterials

## Abstract

In vitro cell culture methods are used extensively to study cellular migration, proliferation, and differentiation, which play major roles in wound healing but the results often do not translate to the in vivo environment. One alternative would be to establish an ex vivo model utilizing human discarded skin to evaluate therapies in a more natural setting. The purpose of this study was to institute such a model by creating ‘wounds’ in the center of a piece of discarded skin and treating them with three different biomaterials: collagen, polyethylene glycol (PEG)-fibrin, or PEG-platelet free plasma (PFP). Explants were cultured for 14 days with supernatant and microscopy images collected every 3 days to assess cytotoxicity and epithelialization. After 14 days, the explants were fixed, sectioned, and stained for cytokeratin-10 (CK-10), alpha-smooth muscle actin (α-SMA), and wheat germ (WG). Compared to controls, similar levels of cytotoxicity were detected for 12 days which decreased slightly at day 14. The PEG-PFP hydrogel-treated wounds epithelialized faster than other treatments at days 6 to 14. A 6-8 cell layer thick CK-10+ stratified epidermis had developed over the PEG-PFP hydrogel and cells co-stained by WG and α-SMA were observed within the hydrogel. An ex vivo model was established that can be used practically to screen different therapies exploring wound healing.

## 1. Introduction

In the United States, it has been estimated that chronic wounds affect more than 6.5 million people [1] while the latest data from National Center for Health Statistics has estimated acute wounds are more prevalent with more than 92 million physician office visits and 41 million requiring emergency department visits in the most recent report from 2015 [2,3]. According to the Food and Drug Administration’s (FDA) guidance on chronic wounds and burns published in 2006, the primary end point for treatments is complete wound closure [4]. To gain FDA approval, potential wound healing products must initially be evaluated for their safety and efficacy using appropriate pre-clinical animal models. However, with a changing regulatory environment on animal studies combined with their cost, other models may be more beneficial in screening therapies prior to the animal evaluation. For instance, a high-throughput laboratory screening tool may facilitate the process to choose the best treatment combination which can then be tested in vivo. Cell culture methods (e.g., in vitro) are well established to evaluate cytotoxicity, biocompatibility, and cell proliferation of wound healing therapies [5,6]. Most of the cell culture methods are two dimensional on plasticware; however, some sophisticated systems are utilizing organotypic three dimensional models that attempt to provide a more natural environment for the cells of interest [7,8]. Still, the in vitro model systems do not recapitulate the complete skin architecture and its micro-environment. To mimic human skin function and physiology, ‘humanized’ mice models were generated [9]. Although humanized mice models have the ability to evaluate therapies of high clinical relevance, the major limitations in their use are the cost and complicated process to ‘mass-produce’ significant quantities of mice for wound-healing studies. Alternatively, human skin samples that are usually discarded after an elective surgical procedure, such as abdominoplasty, may provide an ideal ex vivo platform to conduct high-throughput wound healing studies in the laboratory [7]. When a skin sample is freshly collected, most of the cells are viable and still can respond to an exogenous physiological insult, i.e., a full-thickness excision wound [10,11,12]. Whereas, ex vivo excisions created on a skin sample possess the key cells like keratinocytes, immune cells, fibroblasts, endothelial cells, and smooth muscle cells capable of migrating within the wound bed. Therefore, ex vivo models may be a good ‘high-throughput’ platform to evaluate materials or products that are intended to promote wound healing.

Most of the regenerative skin products on the market are acellular skin substitutes with very few products incorporating therapeutic agents or cells to improve wound healing [13,14,15]. One such line of materials that are currently under investigation are hydrogels. Hydrogels are advantageous in both providing a moist wound environment, as well as acting as a conducive platform for skin cells to migrate and proliferate [16]. Natural biological polymers, such as collagen-, fibrin-, and glycosaminoglycan-based hydrogels have precedence due to their excellent biocompatibility, ability to mimic natural skin architecture/composition, and interaction with cells in vitro and in vivo [17,18,19,20]. Collagen is a well-known protein biopolymer and can be derived from a wide variety of animal tissues to bacterial expression systems [21,22]. One of most abundant tissue sources is tendon; especially rat tail and Achilles [23,24]. Tendon collagen is commonly used for in vitro experiments as well as for tissue engineering purposes because of the molecular composition and homogeneity of the type I collagen extracted [25,26]. Of note, most of the FDA-approved acellular dermal substitutes are based on collagen-based matrices in an attempt to mimic the high collagen content of dermis. Other polymers widely investigated for wound-healing purposes are fibrin-based products (i.e., fibrin sealants) [27]. Similar to collagen, fibrin-based hydrogels are investigated to support cell attachment and proliferation. Fibrin mimics the provisional matrix composition of a wound and provides the natural chemical and physical cues for cells to migrate and proliferate. We have previously shown fibrin hydrogels developed by copolymerizing with polyethylene glycol (PEG) are capable of improving angiogenesis both in vitro and in vivo [28,29,30,31]. Parallel to fibrin, blood plasma has intrinsic fibrinogen along with a plethora of growth factors. Moreover, platelet-rich plasma (PRP) is widely used in clinical practice for tissue repair purposes [32,33]. We recently developed a simple, cost-effective, and clinically usable human platelet-free plasma (PFP)-based hydrogel and demonstrated delivery of adipose-derived stem cells (ASCs) in the PEG-PFP hydrogel improved vasculogenesis in vivo [34]. To this end, induction of angiogenesis is one facet of fibrin and plasma-based hydrogels, while these hydrogels may equally support delivery of therapeutics or re-growth of other skin cells.

In our current study, we have established an ex vivo full-thickness excision wound model using human discarded abdominoplasty skin samples that were collected after informed patient consent for research purposes. We have investigated the ability of collagen, fibrin, and human blood plasma-based hydrogels in promoting keratinocyte migration, proliferation, and differentiation. Specifically, the hydrogel’s ability to promote wound re-epithelialization is thoroughly evaluated.

## 2. Results

### 2.1. Initial 4 mm Pilot Study to Determine Feasibility of Ex Vivo Model

**Figure 1:**
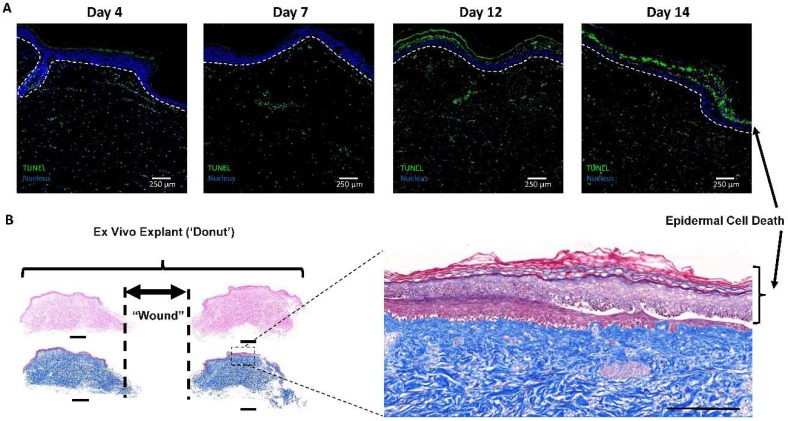
Tissue viability in the 4 mm punch wound pilot study. (**A**) Ex vivo untreated control samples were harvested at the stated time points, sectioned, and stained for cellular death by TUNEL (green staining). A significant increase in terminal deoxynucleotidyl transferase dUTP nick-end labeling (TUNEL) staining was evident in the epidermis over time (white dotted line designates the bottom of the epidermis). (**B**) Representative hematoxylin and eosin (H&E) (left side upper 2 sections) and Masson’s Trichrome (MTC, left side lower 2 sections) staining of day 14 untreated explant cut directly through the center of the donut resulting in 2 pieces with the edges of the wound represented by dotted lines. Scale bars equal 1 mm. Enlarged MTC image to the right indicates similar epidermal cytotoxicity that is observed in the TUNEL staining above. Scale bar equals 250 µm.

De-identified samples were collected from the operating room and processed immediately. The initial attempt at establishing the model was similar to previously reported ex vivo models [10,11,12,35], except we chose to create full-thickness wounds by completely excising the epidermis and dermis from the tissue forming a true ‘donut’. Figure 1A shows cell death increasing via terminal deoxynucleotidyl transferase dUTP nick-end labeling (TUNEL) staining as the explants were cultured for 14 days. In Figure 1B, the epidermis was observed to be separating from the underlying tissue, as reported previously by other researchers with this type of model [10,11,12,35]. However, a new epidermis appears to have formed beneath the ‘old’ dead epidermis. Our goal was to utilize this model as a screening tool to evaluate the wound-healing potential of different biomaterials on actual human tissue. To accomplish this, we chose to first test PEG-PFP hydrogels that have been developed in our laboratory [28].

**Figure 2:**
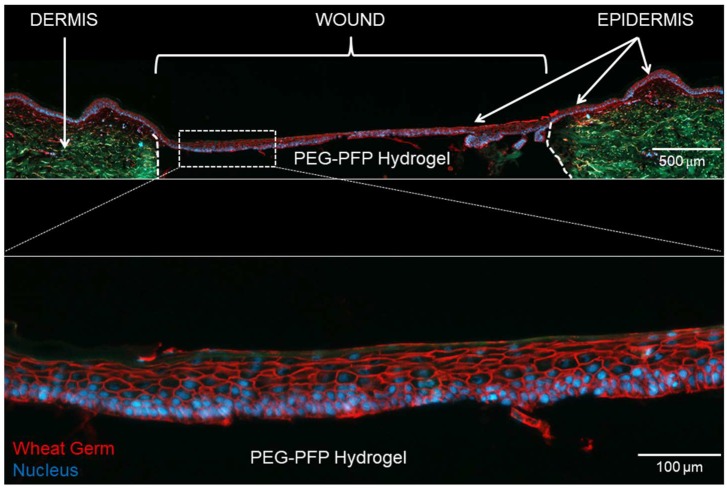
Re-epithelialization of 4 mm ex vivo explant. (**Top**) An explant treated with polyethylene glycol-platelet free plasma (PEG-PFP) hydrogel for 14 days was cryopreserved in a sucrose gradient, sectioned, and stained with wheat germ (WG, red) to visualize all plasma membrane surfaces and counterstained with 4′,6-diamidino-2-phenylindole (DAPI) for nuclei (blue). The white dotted line represents the boundary between normal dermis (green auto-fluorescence) and the wound. (**Bottom**) Enlarged image shows the multilayer epidermis (6–8 cell layers thick) that formed over the PEG-PFP hydrogel.

Figure 2 shows a PEG-PFP treated 4 mm ex vivo explant that was cultured for 14 days. A multilayer (6–8 cell layers thick) epidermis formed and completely re-epithelialized the wound over the top of the PEG-PFP hydrogel. Two issues that arose during this process were the rate of re-epithelialization (<5 days) and the minute volume (35 µL) of the hydrogel leading to inconsistent gelling of the biomaterials. Because of these issues and the promising results seen thus far, we increased the size of the wound to accommodate a larger volume treatment which allows more consistent creation of the hydrogels. It was determined that an 8 mm punch would require ~150 µL to fill the wound and the following results are presented based on that size and volume.

### 2.2. Hydrogel Treatments do not Increase Cell Death in 8 mm Ex Vivo Explants

**Figure 3:**
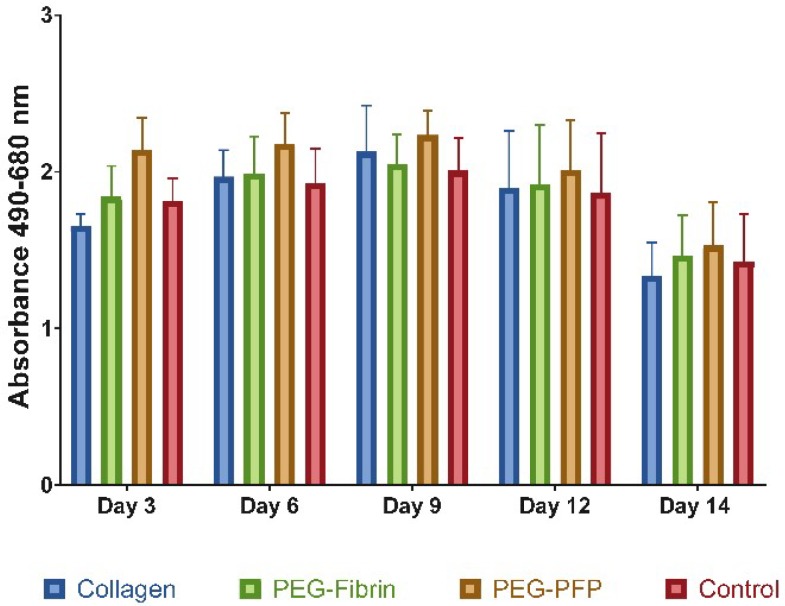
Cytotoxicity analysis using lactate dehydrogenase (LDH) quantification of the supernatant of the treated wounds over time. Media was collected and replaced every 3 days. Cell viability between treatment groups was similar and no statistical differences were observed between any of the treatments or time points (*N* = 5 samples).

Three different biomaterials: collagen, PEG-fibrin, and PEG-PFP were used as treatments to fill the wound in 8 mm ex vivo explants. The samples were cultured for 14 days with the media collected and changed every 3 days. Tissue viability was assessed by the lactate dehydrogenase (LDH) assay. No differences in LDH activity were measured at any time point comparing all treatment groups including control (untreated) wounds (Figure 3). A slight decrease in the overall LDH activity was observed at the day 14 time point but was not statistically significant.

### 2.3. PEG-PFP Hydrogels Increase Wound Closure Rates

**Figure 4:**
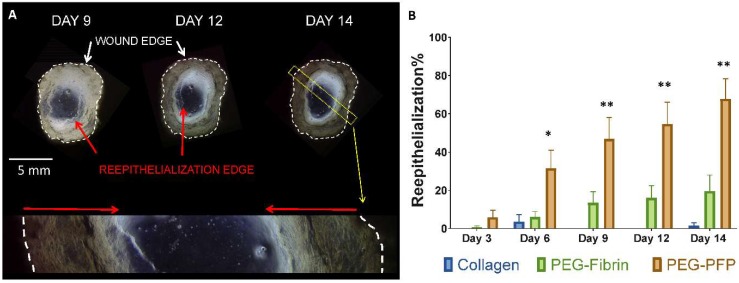
Re-epithelialization of ex vivo explants. (**A**) Representative microscopy images of a PEG-PFP treated wound that were captured over the time course and stitched together to create the above entire view of the ‘wound’. The white dotted lines represent the edges of the actual wound created by the biopsy punch. The red arrows are pointing to the leading edge of the newly formed epidermis. Light does not pass through the full thickness tissue and is reflected by the black regions around each wound. The bottom is an enlarged view of the yellow dotted rectangle from the day 14 image. The newly formed epidermis is highlighted by the red arrows indicating how much has been re-epithelialized by day 14. (**B**) Percent wound closure was quantified for all explants over the time course by measuring the total wound area and the area enclosed by the leading edge of the neo-epidermis. PEG-PFP was significantly different from all other treatment groups. * *p* < 0.01; ** *p* < 0.001, *N* = 5.

Microscopy images were captured at all time points so that the wound closure or re-epithelialization rates could be determined. Figure 4A shows a PEG-PFP hydrogel treated wound at days 9, 12, and 14. The advancing epithelial edge is clearly observed in the digital images with the open area decreasing over time. The enlarged image of day 14 explant (Figure 4A bottom) definitively shows the advancing epithelium, indicated by the red arrows. Significant differences in the re-epithelialization rates were observed comparing the 3 different biomaterials as depicted in Figure 4B. PEG-PFP treated wounds closed at a faster rate than all other wounds from days 6 to 14. (Day 14: 67.8 ± 10.6% vs. 19.6 ± 8.4% vs. 1.6 ± 1.6% for PEG-PFP, PEG-fibrin, and collagen respectively, *p* < 0.0001). While the PEG-fibrin appeared to a better biomaterial than collagen in promoting wound closure at day 14, it was not found to be significant (*p* = 0.0626).

### 2.4. Epidermal Stratification with Polyethylene Glycol-Platelet Free Plasma (PEG-PFP) Hydrogels

**Figure 5:**
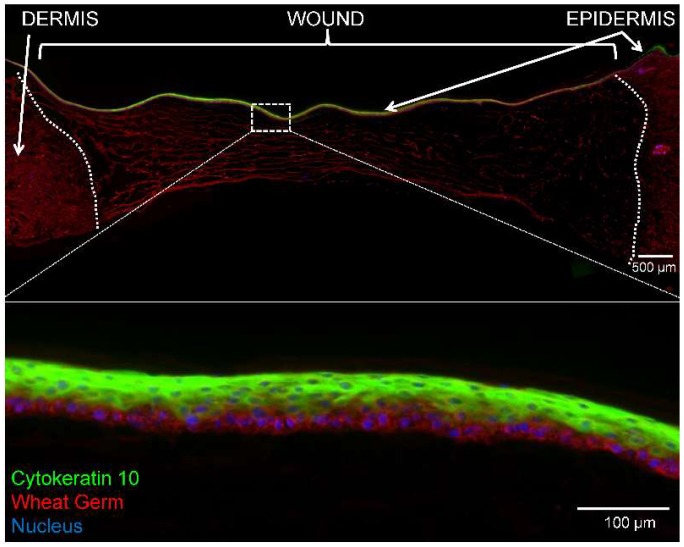
Immunofluorescence of epidermal stratification on day 14 explants treated with PEG-PFP. (**Top**) CK-10 (green) stained the suprabasal layer of cells indicating formation of a stratified epidermis. Sections were counterstained with WG (red) to show the entire newly formed epidermis Nuclei (blue) are observed throughout the PEG-PFP hydrogel-treated area. The white dotted line represents the boundary from the normal dermis and the wound treated with PEG-PFP. (**Bottom**) Enlarged view of white boxed region with a better visual representation of the stratified epidermis from the center of the wound.

In an effort to determine if the newly formed epidermis contained truly stratified epithelial cells, the sections were stained for CK-10 to label the upper suprabasal layer (Figure 5). In the enlarged image in Figure 5, the multilayer epidermis consists of stratified epithelial cells attached to a layer of cells resembling simple basal epithelial cells. In the CK-10 stained areas, the cells are flatter with cuboidal morphology, with the nuclei more horizontally oriented analogous to the normal epidermis.

### 2.5. Cellular Migration and Proliferation with the Hydrogels

**Figure 6:**
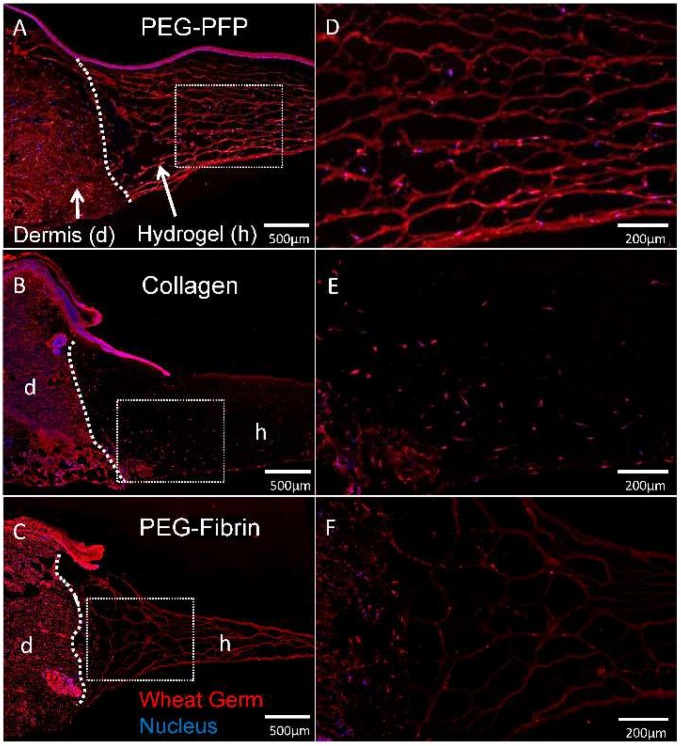
Cellular migration into hydrogels. (**A**–**C**) Explants treated with different hydrogels were cryopreserved after 14 days in culture, sectioned, and stained with wheat germ (WG, red) to visualize all plasma membrane surfaces and counterstained with DAPI for nuclei (blue). Cells originating in the dermis of the explant can be seen infiltrating and migrating into the hydrogels. White dotted line represents the boundary of the normal dermal tissue (d) and hydrogel (h) in the wound. (**D**–**F**) Respective enlarged images of sectioned hydrogels demonstrate cellular density of migrating cells.

At the day 14 time point, there was noticeable/marked degradation of the PEG-fibrin hydrogels compared to the other hydrogels. Cell migration in all the hydrogels were limited to the periphery of the wound edge. Although we did not quantitate the distance of cell migration, wheat germ (WG)-stained sections in Figure 6 indicates more cells have migrated into PEG-PFP hydrogels and collagen than PEG-Fibrin.

### 2.6. α-SMA Positive Cells Infiltrate into the PEG-PFP Hydrogels

**Figure 7:**
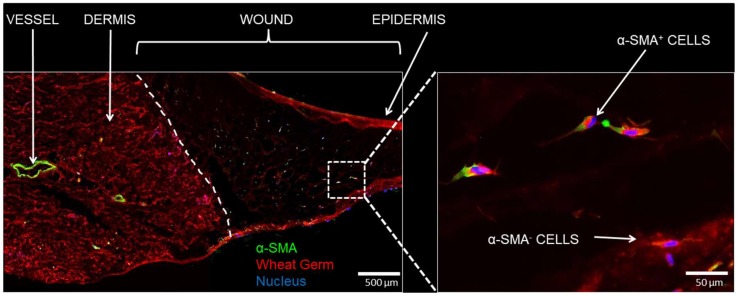
Immunofluorescence of cellular infiltration. (**Left**) day 14 sections treated with PEG-PFP were stained with α-smooth muscle actin (α-SMA, green), a blood vessel and mesenchymal stem cell/myofibroblast marker, and counterstained with WG (red). The white dotted line represents the boundary between normal dermis and the wound treated with PEG-PFP. (**Right**) Enlarged view of white boxed region that shows cells from the normal tissue have infiltrated and proliferated into the hydrogel. Both α-SMA positive and negative stained cells were observed.

The cells that were observed in the PEG-PFP hydrogels were further assessed for their identification. Sections were stained for α-SMA to determine if the nature of the cells observed in the hydrogels were from dermal fibroblasts. Figure 7 shows populations of cells that are both positive and negative for α-SMA.

### 2.7. Human PFP Contains Detectable Levels of Cytokines and Growth Factors

**Figure 8:**
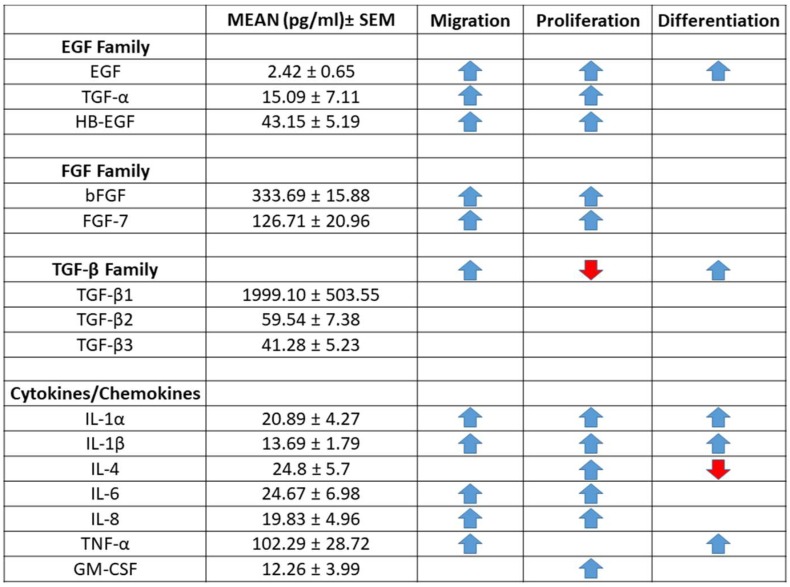
Quantification of cytokines, chemokines, and growth factors in human platelet-free plasma. Analysis of 200 proteins from three patient samples by Q4000 Quantibody^®^ revealed detectable levels of 176 proteins. A selection of proteins and growth factors related to migration, proliferation, and differentiation of keratinocytes are presented as mean ± standard error of the mean (SEM) in pg/mL. Arrow direction, increases (blue) or decreases (red), indicates the effect of the particular protein on either migration, proliferation, and/or differentiation of keratinocytes as reviewed in the literature [36,37,38].

Since the study indicated the PEG-PFP hydrogels can contribute to re-epithelialization, we chose to determine what proteins were present in the PFP. Protein concentrations of 200 cytokine, chemokine, and growth factors on three PFP samples were analyzed by enzyme-linked immunosorbent assay (ELISA) (Table S1 for complete list). Proteins of interest to the migration, proliferation, and differentiation of keratinocytes were detected within the PFP (Figure 8). The epidermal growth factors (EGF), fibroblast growth (FGF), and transforming growth factor (TGF-β) families of proteins are directly involved with the re-epithelialization process and their average levels are reported in pg/mL in Figure 8. Several analytes known to be involved in these processes (IGF-1, IFN-γ, BMP-6) were either not on the arrays or were below the level of detection.

## 3. Discussion

Acute wound care is a major socio-economic problem in the United States. If not appropriately treated, acute wounds may result in long-term healing complications, such as infection, contraction, and scarring, which negatively impact the quality of life of an individual [1]. According to the FDA, complete wound closure is defined as “skin re-epithelialization without drainage or dressing requirements confirmed at two consecutive study visits 2 weeks apart” [4]. From a clinical perspective, complete wound closure is considered ‘The Gold Standard’ to determine success of a treatment.

A variety of next-generation biocompatible wound-healing materials, ranging from immediate temporary wound coverings to complete skin substitutes, with or without active pharmacological agents, are being developed [39,40,41]. It is essential to determine if any of these new wound dressings would meet the FDA standard of inducing successful and complete wound re-epithelialization. Therefore, there is a requirement to screen such products at a reproducible and faster pace by using an economically feasible and clinically valid experimental model. The focus of our current study was to investigate similar materials in a potential high-throughput model prior to investigation in more expensive pre-clinical large animal models. Here, we are exploring the effects of hydrogel-based biomaterials on wound closure using a human ex vivo full-thickness wound model. The currently developed ex vivo model closely mimics any acute injuries such as full-thickness skin abrasions or tears, deep cuts, or excised burn wounds.

Hydrogel-based wound dressings currently have a significant impact on acute wound care due to their ability to provide a moist wound environment, adequate gas and fluid exchange, to support cell growth, and to be capable of delivering therapeutic drugs to the wound bed [42]. Three different hydrogel systems (i.e., collagen, PEG-fibrin, and PEG-PFP) were tested in this study. We initially screened these hydrogels using a 4 mm full-thickness excision wound model. The explants treated with the PEG-PFP hydrogels had a complete and multilayer epidermis. Specifically, the newly formed epithelial layer on the top of the hydrogel has a distinct cuboidal cell morphology, well-defined cell membrane, and centrally located spherical-to-oval nuclei. These are some of characteristic features of the formation of a functionally active epithelia [43]. Even though the 4 mm excision wound model provided evidence for hydrogels to support wound re-epithelialization, the major drawback with the 4 mm wounds was the small volume (~35 µL) of biomaterial required to fill the ‘wound’. This creates reproducibility as well as logistical concerns. As previously stated, the tissue is initially submerged in Hank’s balanced salt solution (HBSS) during preparation and the smallest amount of excess solution could dilute this minute volume even further, thereby contributing to inadequate or no gelation occurring. Another consideration is the viscosity of the biomaterials under investigation. It is logistically difficult to pipette viscous solutions at these tiny volumes consistently. In order to overcome these issues, we then evaluated the hydrogels using an 8 mm full-thickness ex vivo wound which allows a more practical working volume (~150 µL).

Use of a critical size wound to test efficiency of a wound dressing is always preferred regardless of the experimental model. The 8 mm excision wound served as a critically sized wound in our current ex vivo condition. Significant differences in the rate of re-epithelialization was observed over time in different hydrogels. Our results indicate PEG-PFP hydrogels are promoting keratinocyte proliferation, migration, and differentiation faster in comparison to PEG-fibrin and collagen gels. It was surprising to note the collagen hydrogels resulted in slower re-epithelialization although well-established in vitro models use collagen gels to regenerate stratified epidermis [44,45,46,47,48]. The major difference is, in vitro, the keratinocyte cells are evenly distributed and cultured, whilst the ex vivo system relies on epithelial cells to migrate and proliferate from the margin of the wound bed. This mechanism is a major advantage of using the ex vivo wound model because it closely mimics how full-thickness wounds heal in humans. This observation circumvents the major criticism with rodent in vivo wound models where the wound heals via contraction due to the presence of subcutaneous panniculus carnosus muscle that would otherwise facilitate skin healing by both contraction and collagen formation [49,50].

We observed the immunophenotype of a re-epithelialized wound bed on top of the PEG-PFP hydrogels using a keratin specific marker, CK-10. The epithelial cells stained positive to CK-10, which is usually expressed by suprabasal keratinizing cells in a normal epithelial. It is well documented that different types of keratins are synthesized at different stages of cell differentiation [51]. The presence of a CK-10 positive multilayer epidermis confirms PEG-PFP hydrogels are an excellent platform to support formation of a functionally active epithelial wound bed. We then analyzed the proteins that may be responsible for the PEG-PFP to promote faster re-epithelialization than the other hydrogels. The analysis detected several factors present within the plasma, which provides a rich mitogenic environment for the cells to migrate to and proliferate in. Specifically, the transforming growth factor (TGF), epidermal growth factors (EGF), and fibroblast growth (FGF) families of proteins are present at significantly detectable levels (Figure 8). Interestingly, most of the growth factors, like EGF, FGF, and keratinocyte growth factor (KGF)/fibroblast growth factor 7 (FGF-7) are supplemented to propagate keratinocytes in vitro, as well as used in organotypic culture to develop multilayer stratified keratinocytes [52,53]. Collagen and fibrin hydrogels lack these growth factors and, therefore, have fewer mitogenic properties limiting keratinocyte migration and proliferation from the wound edges.

Finally, cell migration within the hydrogels was observed; all of the hydrogel supported some amount of stromal cell migration and proliferation around the periphery of the wound bed. Amongst the different hydrogels used, PEG-PFP was more favorable to cell migration and proliferation and a subset of the migrated cells were α-SMA positive, a marker of stromal cells present around blood vessels and in the dermis. Throughout the course of the experiment, a significant amount of hydrogel shrinkage was observed and we speculate that to be associated to enzymes and cytokines that might have secreted from the ex vivo skin over time. De Wever et al. had clearly shown the ex vivo skin to retain viable T-cells around the native blood vessels and in the dermis, along with increased amounts of matrix metalloproteinase 1 (MMP-1), that might have contributed to the aforementioned observation [7].

Collectively, our data indicates PEG-PFP hydrogels are influencing keratinocytes to proliferate, migrate, and differentiate faster than the other hydrogels evaluated. More importantly, the newly formed epidermis consists of stratified layer of cells with suprabasal keratinocytes in the orientation similar to those observed in normal skin. There are certain limitations to this study, which include: (i) the tissue obtained was from elective abdominoplasty surgeries. The patients ranged in age from 34–66 and had a body mass index (BMI) of 17–42. Excess abdominoplasty tissue is inherently not normal skin and the variability we observed in the explants confirmed this observation; and (ii) the current ex vivo model cannot recapitulate the inflammatory responses that is observed in an in vivo animal model. However, the model can be used to test products that are capable of promoting wound epithelialization, pigmentation, hair-follicle regeneration, vascularization, and dermal reorganization. Ultimately, biomaterials can be utilized as a delivery device for therapeutics like antibiotics, immune modulatory factors, and cell therapies (i.e., stem cells, keratinocytes, and/or fibroblasts) and evaluated in the ex vivo system.

## 4. Materials and Methods

### 4.1. Ex Vivo Model

De-identified human abdominal skin and underlying adipose tissue was obtained from elective abdominoplasty surgeries with appropriate written consent. This study was conducted under a protocol reviewed and approved by the US Army Medical Research and Materiel Command Institutional Review Board and in accordance with the approved protocol (H-11-020/M-10128; initial approval 27 June 2011). A total of 8 samples were utilized for these experiments (*N* = 2 for 4 mm feasibility and *N* = 6 for 8 mm wound study). Tissue samples were then washed with HBSS and 1X antibiotic-antimycotic (ABAM, 100 U/mL of penicillin G, 100 μg/mL of streptomycin sulfate, and 0.25 μg/mL of amphotericin B) to disinfect the tissue. Using sterile surgical instruments, the excessive subdermal adipose tissue was removed from the overlying epidermis and dermis of the abdominal skin. A 4–8 mm punch biopsy was used to create an artificial full thickness wound within the tissue. Surgical scissors were used to trim excess tissue around the wounds, eventually creating an annular cylinder (i.e., a ‘donut’). Individual skin explants were placed in cell culture inserts in a six-well plate. The explants were cultured in a 5% CO_2_ humidified incubator at 37 °C in “growth media”, MesenPRO RS™ supplemented with MesenPRO RS™ growth supplement, ABAM, and 2 mM of L-glutamine (Life Technologies, Carlsbad, CA, USA). The growth media was added to the bottom of the plate and within the insert up to the epidermal–dermal boundary so that the epidermis of the explants was exposed to the air. Media was collected during media changes that occurred every 3 days and stored at −80 °C for later LDH analysis.

### 4.2. Platelet-Free Plasma Preparation

Whole blood was collected from volunteers at the USAISR blood bank in tubes containing 3.2% buffered sodium citrate solution (BD Falcon). The blood was initially centrifuged at 800× *g* for 10 min at 4 °C with no brake. The plasma fraction (upper ~2/3) of the tubes was transferred to 50 mL conical tubes and centrifuged again at 4300× *g* with no brake to remove any residual platelets. The supernatant (platelet-free plasma, PFP) was aliquoted into 15 mL tubes and stored at −20 °C until further use.

### 4.3. Quantification of Chemokines in Human PFP

The concentration of analytes in three PFP samples were determined using the Quantibody^®^ Multiplex ELISA array (Q4000, RayBiotech, Norcross, GA, USA) according to manufacturer’s instructions. Briefly, the glass slides provided by RayBiotech were allowed to equilibrate to room temperature (RT) and air dry for 2 h. Wells were blocked with sample diluent for 30 min at RT on a rocker. Standards were prepared as indicated while samples were diluted 1:1 in diluent buffer and incubated overnight at 4 °C with mild shaking. Slides were washed, incubated with biotinylated antibody cocktail at RT for 2 h, washed, incubated with Cy3 equivalent dye-streptavidin at RT for 1 h, washed, dried, and read on an InnoScan 710 microarray scanner (Innopsys, Carbonne, France). Data was extracted and processed using the supplied Quantibody^®^ Q-Analyzer software (version 8.10.4; RayBiotech, Norcross, GA, USA). Standard curves were created for each analyte. The median fluorescent signal (532 nm) for each analyte was adjusted to background intensity and the concentration was determined based on its respective standard curve. The concentration of each analyte was averaged for the three samples.

### 4.4. Hydrogel Formulations

#### 4.4.1. Collagen Hydrogel

Collagen hydrogels were prepared within the biopsy punch of the skin explants using previously published protocols [54]. Briefly, Dulbecco’s phosphate buffered saline (Sigma-Aldrich, St. Louis, MO, USA) and 1 N NaOH were added to type 1 rat tail collagen (5 mg/mL, Trevigen, Gaithersburg, MD, USA) to achieve a final collagen concentration of 4.5 mg/mL at a pH of 6.8 to 7.0. Collagen gels were allowed to polymerize for 30 to 40 min in a 5% CO_2_ humidified incubator at 37 °C.

#### 4.4.2. PEG-Fibrin Hydrogel

PEG-fibrin hydrogels were prepared within the biopsy punch of the skin explants as previously described [55]. Briefly, modified PEG (succinimidyl glutarate-modified polyethylene glycol; SG-PEG-SG, 3400 Da; NOF America Corporation, White Plains, NY, USA) was dissolved in tris-buffered saline (TBS, pH 7.8, Sigma-Aldrich) at a stock concentration of 8 mg/mL and filter sterilized with a 0.22 µm filter. The PEG solution was added to 40 mg/mL fibrinogen (Sigma-Aldrich) at concentration ratio of 1:10 and incubated for 20 min in a 5% CO_2_ humidified incubator at 37 °C. An equal volume of thrombin (Sigma-Aldrich) in 40 mM CaCl_2_ at a stock concentration of 25 U/mL was added to the PEG-fibrinogen solution and incubated for 10 min at 37 °C to allow for complete polymerization/gelation.

#### 4.4.3. PEG-PFP Hydrogel

PEG-PFP hydrogels were prepared within the biopsy punch of the skin explants as previously described [28]. Briefly, SG-PEG-SG was dissolved in TBS at a stock concentration of 8 mg/mL and filter sterilized with a 0.22 µm filter. The PEG solution was added to thawed PFP at a concentration ratio of 1:10 and incubated for 20 min in a 5% CO_2_ humidified incubator at 37 °C. A 10% volume of thrombin in 40 mM CaCl_2_ at a stock concentration of 100 U/mL was added to the PEG-PFP solution and incubated for 10 min at 37 °C to allow for complete polymerization/gelation.

### 4.5. Tissue Viability

The viability of the explants over time was assessed by the LDH assay according to manufacturer’s instructions (Pierce LDH assay kit, Thermo-Fisher, Waltham, MA, USA). Cell culture media (supernatant) from the explants was collected at time of media change in 96-well plates. Tetrazolium test mix was added to each well and incubated in the dark for 30 min at room temperature (RT) with gentle shaking. Absorbance at OD 490 nm was measured with a spectrophotometer and corrected for background absorbance (OD 680 nm).

### 4.6. Microscopy Documentation of Wound Healing

Microscopy digital images were captured over time using a Zeiss inverted microscope (Carl Zeiss, Thornwood, NY, USA). Individual images were stitched together to form a single montage image of the complete wound using Research Image Composite Editor (Microsoft Corporation, Redmond, WA, USA). Image J 1.45s (National Institutes of Health, Bethesda, MD, USA) software was used to measure the total wound size and the ‘open’ wound by tracing the leading epithelial edge; percent of wound closure was calculated by 100% (open wound area/total wound area* 100).

### 4.7. Immunohistochemistry

Untreated explants were fixed in 10% buffered formalin, processed, paraffin embedded, sectioned at 5 µm, stained with TUNEL, H&E, and Masson’s trichrome, and imaged using a Zeiss inverted microscope. Ex vivo explants treated with hydrogels were cryopreserved and embedded using a gradient sucrose technique [56]. Explants were washed with HBSS, fixed with 4% paraformaldehyde (PFA, EMS, Hatfield, PA, USA) for 20 min, and exposed to increasing concentrations of sucrose (5–20%, 30 min each), and incubated overnight with 20% sucrose-Histoprep (Fisher Scientific, Pittsburgh, PA, USA) mixture and frozen by liquid nitrogen cooled isopentane. Cryosections (6 µm) were cut using a cryostat (Leica Microsystems, Wetzlar, Germany), washed with HBSS, and fixed with 4% PFA for 20 min. Sections were blocked by incubating for 1 h with 5% donkey serum in HBSS or with 1% bovine serum albumin in HBSS containing 0.01% Triton X-100 and then washed with HBSS. Sections were stained with anti-human primary antibodies (Abcam, Cambridge, UK) cytokeratin 10 (CK-10, 1:50) to detect the suprabasal epidermis and alpha-smooth muscle actin (α-SMA, 1:100) to label infiltrating cells that have grown into the hydrogels at 4 °C overnight. Sections were washed with HBSS and incubated with host species-specific Alexa Fluor 647 secondary antibodies (Life Technologies) for 45 min at RT. Samples were counterstained with rhodamine wheat germ (WG, 1:500, Vector Labs, Burlingame, CA, USA) for 1 h at RT and mounted using Prolong Gold with DAPI (Thermo). Fluorescence images were captured using a Zeiss inverted microscope, with the WG captured at 545 nm, CK-10 and α-SMA captured at 647 nm to avoid signal disruption from auto-fluorescence from dermal collagen.

### 4.8. Statistical Analysis

For all experiments, statistically significant differences were determined with two-way analysis of variance (ANOVA) with Tukey’s post-hoc test for multiple comparisons using GraphPad Prism 7.01 (GraphPad Software Inc, La Jolla, CA, USA). All data is reported as the mean ± SEM of at least 3 independent explants for each donor sample. Statistical significance was found when *p* < 0.05 and is indicated in the figures.

## 5. Conclusions

A practical 3D ex vivo skin model to align with the FDA’s required primary wound-healing endpoint is superior to other standard in vitro cell cultures and existing ex vivo models. This study establishes an ex vivo human skin wound model that will act as a prototype to investigate biomaterials specifically developed to improve wound healing. Here, we report the successful evaluation of PEG-PFP hydrogels as a potential therapy in promoting wound closure. This model can potentially be used as a screening tool to study wound healing in order to minimize the number of animals used in research.

## Supplementary Materials

Supplementary materials can be found at http://www.mdpi.com/1422-0067/19/10/3156/s1.

## Abbreviations

PEG	Polyethylene glycol
PFP	Platelet-free plasma
CK-10	Cytokeratin 10
α-SMA	Alpha smooth muscle actin
WG	Wheat germ
FDA	Food and Drug Administration
PRP	Platelet-rich plasma
ASCs	Adipose derived stem cells
HBSS	Hank’s balanced salt solution
ABAM	Antibiotic-antimycotic
LDH	Lactate dehydrogenase
RT	Room temperature
H&E	Hematoxylin and eosin
PFA	Paraformaldehyde
TUNEL	Terminal deoxynucleotidyl transferase dUTP nick-end labeling
TGF	Transforming growth factor
EGF	Epidermal growth factor
KGF	Keratinocyte growth factor
FGF	Fibroblast growth factor

## References

[1] Sen C.K., Gordillo G.M., Roy S., Kirsner R., Lambert L., Hunt T.K., Gottrup F., Gurtner G.C., Longaker M.T. (2009). Human skin wounds: A major and snowballing threat to public health and the economy. Wound Repair Regen..

[2] (2015). National Center for Health Statistics; National Ambulatory Medical Care Survey: 2015 State and National Summary Tables. https://www.cdc.gov/nchs/data/ahcd/namcs_summary/2015_namcs_web_tables.pdf.

[3] National Center for Health Statistics (2015). National Hospital Ambulatory Medical Care Survey: 2015 Emergency Department Summary Tables. https://www.cdc.gov/nchs/data/nhamcs/web_tables/2015_ed_web_tables.

[4] U.S. Department of Health and Human Services, Food and Drug Administration (2006). Guidance for Industry Chronic Cutaneous Ulcer and Burn Wounds—Developing Products for Treatment. https://www.fda.gov/downloads/drugs/guidances/ucm071324.pdf.

[5] Li W., Zhou J., Xu Y. (2015). Study of the in vitro cytotoxicity testing of medical devices. Biomed. Rep..

[6] Williams D.F. (2008). On the mechanisms of biocompatibility. Biomaterials.

[7] De Wever B., Kurdykowski S., Descargues P. (2015). Human Skin Models for Research Applications in Pharmacology and Toxicology: Introducing NativeSkin^®^, the “Missing Link” Bridging Cell Culture and/or Reconstructed Skin Models and Human Clinical Testing. Appl. In Vitro Toxicol..

[8] Shamir E.R., Ewald A.J. (2014). Three-dimensional organotypic culture: Experimental models of mammalian biology and disease. Nat. Rev. Mol. Cell Biol..

[9] Salgado G., Ng Y.Z., Koh L.F., Goh C.S.M., Common J.E. (2017). Human reconstructed skin xenografts on mice to model skin physiology. Differentiation.

[10] Hodgkinson T., Bayat A. (2015). Ex vivo evaluation of acellular and cellular collagen-glycosaminoglycan flowable matrices. Biomed. Mater..

[11] Hodgkinson T., Bayat A. (2016). In vitro and ex vivo analysis of hyaluronan supplementation of Integra(R) dermal template on human dermal fibroblasts and keratinocytes. J. Appl. Biomater. Funct. Mater..

[12] Hodgkinson T., Yuan X.F., Bayat A. (2014). Electrospun silk fibroin fiber diameter influences in vitro dermal fibroblast behavior and promotes healing of ex vivo wound models. J. Tissue Eng..

[13] Lazic T., Falanga V. (2011). Bioengineered skin constructs and their use in wound healing. Plast. Reconstr. Surg..

[14] Rizzi S.C., Upton Z., Bott K., Dargaville T.R. (2010). Recent advances in dermal wound healing: Biomedical device approaches. Expert Rev. Med. Device.

[15] Stone Ii R., Natesan S., Kowalczewski C.J., Mangum L.H., Clay N.E., Clohessy R.M., Carlsson A.H., Tassin D.H., Chan R.K., Rizzo J.A. (2018). Advancements in Regenerative Strategies Through the Continuum of Burn Care. Front. Pharmacol..

[16] Madaghiele M., Demitri C., Sannino A., Ambrosio L. (2014). Polymeric hydrogels for burn wound care: Advanced skin wound dressings and regenerative templates. Burns Trauma.

[17] Bano I., Arshad M., Yasin T., Ghauri M.A., Younus M. (2017). Chitosan: A potential biopolymer for wound management. Int. J. Biol. Macromol..

[18] Catanzano O., D’Esposito V., Acierno S., Ambrosio M.R., De Caro C., Avagliano C., Russo P., Russo R., Miro A., Ungaro F. (2015). Alginate-hyaluronan composite hydrogels accelerate wound healing process. Carbohydr. Polym.

[19] Naseri-Nosar M., Ziora Z.M. (2018). Wound dressings from naturally-occurring polymers: A review on homopolysaccharide-based composites. Carbohydr. Polym.

[20] Smith M.M., Melrose J. (2015). Proteoglycans in Normal and Healing Skin. Adv. Wound Care (New Rochelle).

[21] Sorushanova A., Delgado L.M., Wu Z., Shologu N., Kshirsagar A., Raghunath R., Mullen A.M., Bayon Y., Pandit A., Raghunath M. (2018). The Collagen Suprafamily: From Biosynthesis to Advanced Biomaterial Development. Adv. Mater..

[22] Tziveleka L.A., Ioannou E., Tsiourvas D., Berillis P., Foufa E., Roussis V. (2017). Collagen from the Marine Sponges Axinella cannabina and Suberites carnosus: Isolation and Morphological, Biochemical, and Biophysical Characterization. Mar. Drugs.

[23] Fauzi M.B., Lokanathan Y., Aminuddin B.S., Ruszymah B.H.I., Chowdhury S.R. (2016). Ovine tendon collagen: Extraction, characterisation and fabrication of thin films for tissue engineering applications. Mater. Sci. Eng..

[24] Rittie L. (2017). Type I Collagen Purification from Rat Tail Tendons. Methods Mol. Biol..

[25] Chattopadhyay S., Raines R.T. (2014). Review collagen-based biomaterials for wound healing. Biopolymers.

[26] Ramshaw J.A. (2016). Biomedical applications of collagens. J. Biomed. Mater. Res..

[27] Foster K. (2007). The use of fibrin sealant in burn operations. Surgery.

[28] Burmeister D.M., Roy D.C., Becerra S.C., Natesan S., Christy R.J. (2018). In Situ Delivery of Fibrin-Based Hydrogels Prevents Contraction and Reduces Inflammation. J. Burn Care Res..

[29] Burmeister D.M., Stone R., Wrice N., Laborde A., Becerra S.C., Natesan S., Christy R.J. (2018). Delivery of Allogeneic Adipose Stem Cells in Polyethylene Glycol-Fibrin Hydrogels as an Adjunct to Meshed Autografts After Sharp Debridement of Deep Partial Thickness Burns. Stem Cells Transl. Med..

[30] Natesan S., Zamora D.O., Wrice N.L., Baer D.G., Christy R.J. (2013). Bilayer hydrogel with autologous stem cells derived from debrided human burn skin for improved skin regeneration. J. Burn Care Res..

[31] Natesan S., Zhang G., Baer D.G., Walters T.J., Christy R.J., Suggs L.J. (2011). A bilayer construct controls adipose-derived stem cell differentiation into endothelial cells and pericytes without growth factor stimulation. Tissue Eng. Part A.

[32] Eppley B.L., Pietrzak W.S., Blanton M. (2006). Platelet-rich plasma: A review of biology and applications in plastic surgery. Plast. Reconstr. Surg..

[33] Kazakos K., Lyras D.N., Verettas D., Tilkeridis K., Tryfonidis M. (2009). The use of autologous PRP gel as an aid in the management of acute trauma wounds. Injury.

[34] Aurora A., Wrice N., Walters T.J., Christy R.J., Natesan S. (2018). A PEGylated platelet free plasma hydrogel based composite scaffold enables stable vascularization and targeted cell delivery for volumetric muscle loss. Acta Biomater..

[35] Balaji S., Moles C.M., Bhattacharya S.S., LeSaint M., Dhamija Y., Le L.D., King A., Kidd M., Bouso M.F., Shaaban A. (2014). Comparison of interleukin 10 homologs on dermal wound healing using a novel human skin ex vivo organ culture model. J. Surg. Res..

[36] Hanel K.H., Cornelissen C., Luscher B., Baron J.M. (2013). Cytokines and the skin barrier. Int. J. Mol. Sci..

[37] Pastar I., Stojadinovic O., Yin N.C., Ramirez H., Nusbaum A.G., Sawaya A., Patel S.B., Khalid L., Isseroff R.R., Tomic-Canic M. (2014). Epithelialization in Wound Healing: A Comprehensive Review. Adv. Wound Care (New Rochelle).

[38] Raja, Sivamani K., Garcia M.S., Isseroff R.R. (2007). Wound re-epithelialization: Modulating keratinocyte migration in wound healing. Front. Biosci..

[39] Boateng J., Catanzano O. (2015). Advanced Therapeutic Dressings for Effective Wound Healing—A Review. J. Pharm. Sci..

[40] Moore A.L., Marshall C.D., Longaker M.T. (2017). Minimizing Skin Scarring through Biomaterial Design. J. Funct. Biomater..

[41] Saghazadeh S., Rinoldi C., Schot M., Kashaf S.S., Sharifi F., Jalilian E., Nuutila K., Giatsidis G., Mostafalu P., Derakhshandeh H. (2018). Drug delivery systems and materials for wound healing applications. Adv. Drug Deliv. Rev..

[42] Francesko A., Petkova P., Tzanov T. (2017). Hydrogel Dressings for Advanced Wound Management. Clin. Med. Chem..

[43] Fuchs E., Raghavan S. (2002). Getting under the skin of epidermal morphogenesis. Nat. Rev..

[44] Sun T.T., Green H. (1976). Differentiation of the epidermal keratinocyte in cell culture: Formation of the cornified envelope. Cell.

[45] Anacker D., Moody C. (2012). Generation of organotypic raft cultures from primary human keratinocytes. J. Vis. Exp..

[46] Boyce S.T., Rice R.K., Lynch K.A., Supp A.P., Swope V.B., Kagan R.J., Supp D.M. (2012). Assessment of replication rates of human keratinocytes in engineered skin substitutes grafted to athymic mice. Wound Repair Regen..

[47] Boyce S.T., Kagan R.J., Greenhalgh D.G., Warner P., Yakuboff K.P., Palmieri T., Warden G.D. (2006). Cultured skin substitutes reduce requirements for harvesting of skin autograft for closure of excised, full-thickness burns. J. Trauma.

[48] Rasmussen C.A., Gibson A.L., Schlosser S.J., Schurr M.J., Allen-Hoffmann B.L. (2010). Chimeric composite skin substitutes for delivery of autologous keratinocytes to promote tissue regeneration. Ann. Surg..

[49] Abdullahi A., Amini-Nik S., Jeschke M.G. (2014). Animal models in burn research. Cell. Mol. Life Sci..

[50] Dorsett-Martin W.A. (2004). Rat models of skin wound healing: A review. Wound Repair Regen..

[51] Steinert P.M. (1995). A model for the hierarchical structure of the human epidermal cornified cell envelope. Cell Death Differ..

[52] Gangatirkar P., Paquet-Fifield S., Li A., Rossi R., Kaur P. (2007). Establishment of 3D organotypic cultures using human neonatal epidermal cells. Nat. Protoc..

[53] Rasmussen C., Thomas-Virnig C., Allen-Hoffmann B.L. (2013). Classical human epidermal keratinocyte cell culture. Methods Mol. Biol..

[54] Helary C., Zarka M., Giraud-Guille M.M. (2012). Fibroblasts within concentrated collagen hydrogels favour chronic skin wound healing. J. Tissue Eng. Regen. Med..

[55] Zhang G., Wang X., Wang Z., Zhang J., Suggs L. (2006). A PEGylated fibrin patch for mesenchymal stem cell delivery. Tissue Eng..

[56] Barthel L.K., Raymond P.A. (1990). Improved method for obtaining 3-microns cryosections for immunocytochemistry. J. Histochem. Cytochem..

